# Supplementary material for: Efficacy and Safety of Sofosbuvir/Velpatasvir Plus Ribavirin in Patients with Hepatitis C Virus-Related Decompensated Cirrhosis
Source: Viruses. 2023 Sep 29;15(10):2026. doi: 10.3390/v15102026 (PMC10611233; doi:10.3390/v15102026)
Supplement: Supplementary file 1 [file viruses-15-02026-s001.zip › viruses-2552454-supplementary.pdf]

Table S1. Deaths.

Age	CTP at baseline, class (score)	MELD score at baseline	Completed treatment	Post-treatment study day of death	Cause of death†	Comment	SVR4	SVR12	SVR24
59	C (10)	11	No*	N/A	Colitis	Presented with colitis and pus pockets. Went on to develop sepsis and renal failure and died. The subject had an ongoing SAE of sepsis at the time of death.	No‡	No‡	No‡
64	C (13)	24	No*	N/A	Probable sepsis with multi-organ failure	Presented with shortness of breath, developed respiratory distress and died of probable sepsis with multiorgan failure. The subject had an ongoing SAE of respiratory distress.	No‡	No‡	No‡
48	B (9)	18	Yes	103	Acute pancreatitis	Had a preventive embolization of a pancreatic aneurysm, which was complicated by acute pancreatitis that led to multiorgan failure and death. The subject had an ongoing SAE of acute pancreatitis.	Yes	Yes	Yes‡
59	B (9)	14	Yes	164	Variceal hemorrhage	Found bleeding and deceased at home. The subject had an ongoing SAE of bleeding varicose veins.	Yes	Yes	Yes‡
58	C (10)	15	Yes	53	Cardiac arrest	Presented with hematemesis and shortness of breath and died of cardiac arrest due to hypovolemic shock because of upper gastrointestinal bleed. The subject had an ongoing SAE of upper gastrointestinal hemorrhage.	Yes	No‡	No‡
53	C (11)	17	Yes	123	Liver failure	Had two admissions for hepatic hydrothorax and later died of liver	Yes	No‡	No‡

						failure. The subject had ongoing SAEs of hepatic hydrothorax and liver failure.			
57	C (11)	17	Yes	90	Liver failure	Diagnosed with infiltrative and metastatic HCC and was not a candidate for treatment. Died of liver failure. The subject had ongoing SAEs of HCC and liver failure.	Yes	No‡	No‡
58	C (11)	15	Yes	80	Sepsis	Presented with ascites and impaired breathing and died of sepsis. The subject had an ongoing SAE of ascites.	Yes	No‡	No‡

All the patients who died were male. *Died 6 and 3 days after treatment initiation respectively; †No deaths were related to treatment; ‡Imputed value. CTP, Child–Turcotte–Pugh; MELD, Model for End-stage Liver Disease; N/A, not applicable; SAE, serious adverse event; SVR4, sustained virologic response after 4 weeks post treatment; SVR12, sustained virologic response after 12 weeks post treatment; SVR24, sustained virologic response after 24 weeks post treatment.

Table S2. Baseline characteristics of subjects who achieved SVR24 and those who did not achieve SVR24.

	**No SVR24*** ***N* = 7**	**SVR24†** ***N* = 24**
Mean age, years (range)	58 (53–64)	54 (39–67)
Male, *n* (%)	7 (100.0)	18 (75.0)
Race, *n* (%)		
White	3 (42.9)	14 (58.3)
Black	2 (28.6)	4 (16.7)
Other/not stated	2 (28.6)	6 (25.0)
HCV GT, *n* (%)		
1a	5 (71.4)	11 (45.8)
1b	0 (0.0)	2 (8.3)
2	0 (0.0)	4 (16.7)
3	2 (28.6)	5 (20.8)
Indeterminate‡	0 (0.0)	2 (8.3)
CTP class, *n* (%)		
B (7–9)	1 (14.3)	8 (33.3)
C (10–15)	6 (85.7)	16 (66.7)
MELD score, *n* (%)		
10–15	4 (57.1)	9 (37.5)
16–20	2 (28.6)	14 (58.3)
21–25	1 (14.3)	1 (4.2)
Ascites, *n* (%)		
None	0 (0.0)	3 (12.5)
Mild/moderate	4 (57.1)	16 (66.7)
Severe	3 (42.9)	5 (20.8)
Encephalopathy, *n* (%)		
None	0 (0.0)	5 (20.8)
Medication-controlled	7 (100.0)	19 (79.2)
HCV RNA, $\log_{10}$ IU/mL mean (SD)	5.0 (0.7)	5.2 (1.3)
HCV RNA ≥800,000 IU/mL, *n* (%)	1 (14.3)	7 (29.2)
HCV treatment experienced, *n* (%)§	0 (0.0)	4 (16.7)
$eGFR_{CG}$, mL/min, mean (SD)	120.9 (47.3)	111.5 (41.9)
AST, U/L, mean (SD)	102.0 (63.7)	102.0 (64.7)
ALT, U/L, mean (SD)	53.6 (35.9)	50.3 (36.4)
Platelets, $\times 10^3/\mu L$, mean (SD)	83.9 (30.8)	92.3 (31.3)
Albumin, g/dL, mean (SD)	2.7 (0.5)	2.8 (0.5)
INR, mean (SD)	1.5 (0.3)	1.5 (0.3)
Hemoglobin, g/dL, mean (SD)	12.1 (1.3)	11.9 (1.3)
Lymphocytes, $\times 10^3/\mu L$ (SD)	1.3 (0.9)	1.8 (0.9)
Bilirubin, mg/dL, mean (SD)	3.5 (1.5)	3.4 (1.6)

*Treatment-emergent deaths *n* = 2; non-treatment-emergent deaths *n* = 4; relapse *n* = 1. The patient who discontinued treatment due to investigator discretion has been excluded; †SVR24 was imputed for two patients who died after achieving SVR12, at post-treatment study day 103 and 164 respectively; ‡Viral load too low to assess; §Treatment-experienced subjects must have completed their most recent HCV treatment at least 8 weeks prior to screening. ALT, alanine aminotransferase; AST, aspartate aminotransferase; CTP, Child–Turcotte–Pugh; $eGFR_{CG}$, estimated glomerular filtration rate by Cockcroft-Gault formula; GT, genotype; HCV, hepatitis C virus; INR, international normalized ratio; IU, international units; MELD, Model for End-stage Liver Disease; SD, standard deviation; SVR24, sustained virologic response after 24 weeks post treatment.

Table S3. Relapse.

Gender	Age	Genotype	Completed treatment	Adherence rate	HCV RNA (IU/mL)							
					Day 1	Week 2	Week 4	Week 8	Week 12	PT Week 4	PT Week 12	PT Week 24
Male	60	3a	Yes	100%	45500	<LLOQ	<15	<LLOQ	<LLOQ	51	<15	9740

HCV, hepatitis C virus; IU, international units; <LLOQ, less than the lower limit of quantitation; PT, post treatment.

Table S4. Change in HRQoL in patients with improved or not improved CTP class between baseline and post-treatment Week 12.

Change between baseline and post-treatment Week 12	Improved *N* = 15	Not improved *N* = 3
CTP class, mean (SD)	−2 (1.3)	0 (0.0)
SF-36		
Physical component score, mean (SD)	-0.3 (8.5)	−9.2 (7.3)
Mental component score, mean (SD)	0.4 (8.9)	−4.4 (7.5)
CLDQ-HCV overall score, mean (SD)	0.5 (0.8)	−0.6 (0.7)
FACIT-F overall score, mean (SD)	4.3 (16.8)	−10.8 (18.8)

Only 2 subjects completed the WPAI: Hep C percent overall work impairment due to HCV questionnaire so the results are not included here. Excludes patients who received LT (*n* = 3) and patients with no baseline or post-treatment Week 12 CTP score or HRQoL data. CLDQ-HCV, Chronic Liver Disease Questionnaire-Hepatitis C; CTP, Child–Turcotte–Pugh; FACIT-F, Functional Assessment of Chronic Illness Therapy-Fatigue; HRQoL, health-related quality of life; LT, liver transplant; SD, standard deviation; SF-36, 36-Item Short Form Survey; WPAI: Hep C, Work Productivity and Activity Impairment: Hepatitis C.
